# Characterization of a novel cysteine protease inhibitor in *Baylisascaris schroederi* migratory larvae and its role in regulating mice immune cell response

**DOI:** 10.3389/fimmu.2022.894820

**Published:** 2022-08-29

**Authors:** Jingyun Xu, Xiaobin Gu, Yue Xie, Ran He, Jing Xu, Lang Xiong, Xuerong Peng, Guangyou Yang

**Affiliations:** ^1^ Department of Parasitology, College of Veterinary Medicine, Sichuan Agricultural University, Wenjiang, China; ^2^ Department of Chemistry, College of Life and Basic Science, Sichuan Agricultural University, Wenjiang, China

**Keywords:** *Baylisascaris schroederi*, cysteine protease inhibitor, monocyte-derived macrophages, CD4+ T cells, TLR signaling pathway

## Abstract

*Baylisascaris schroederi* (*B. schroederi*) is a severe threat to the survival of giant pandas. Currently, the immune regulation mechanism of *B. schroederi* is poorly understood. Cysteine protease inhibitors (CPI) play important roles in the regulation of host immune responses against certain nematodes. In this study, a recombinant CPI of *B. schroederi* migratory larvae (rBsCPI-1) was cloned and expressed, and the effects of rBsCPI-1 on the physiological activities and antigen presentation of monocyte-derived macrophages (MDMs) were analyzed. We also analyzed the regulatory effects of rBsCPI-1 on the proliferation and differentiation of CD4+ T cells. And further identified the signaling pathways which play important roles in this process. The results showed that rBsCPI-1 activated the TLR2/4-small Rho GTPases-PAK1 pathway. On the one hand, it increased the phagocytosis and migration of MDMs. On the other hand, it activated downstream MAPK and NF-κB signaling pathways to induce apoptosis of MDMs. rBsCPI-1 also induced MDMs to polarize to the M2 subtype, thereby exerting an immunosuppressive effect. Meanwhile, rBsCPI-1 inhibited the antigen presentation process by decreasing the expression of MHC-II molecules, further inhibiting the proliferation of CD4+ T cells and inducing a Th1/Th2 mixed immune response. Treg cells with immunosuppressive effects were increased. The PD-L2/PD-1 and CD80/CTLA-4 signaling pathways between MDMs and CD4+ T cells were also activated by rBsCPI-1. In conclusion, this study preliminarily confirmed that rBsCPI-1 affects the physiological activities and polarization of MDMs through the TLR2/4 signaling pathway, and further interferes with antigen presentation response, inducing CD4+ T cells to play an immunosuppressive cellular response during the migratory process of *B. schroederi*. Thus, this study will provide a reference for elucidating the immune evasion mechanism of *B. schroederi* and developing new drugs and protective vaccines against *B. schroederi*.

## Introduction

As members of a rare and endangered wild species unique to China, giant pandas (*Ailuropoda melanoleuca*) are known as “national treasures” and “living fossils” (1). Giant pandas are obligate hosts of *Baylisascaris schroederi* (*B. schroederi*), and infection with *B. schroederi* is very common (1, 2). The parasitism of *B. schroederi* usually leads to the death of wild and captive giant pandas (1–3). *B. schroederi* migration larvae can migrate to various organs of giant pandas, inducing “visceral larva migrans (VLM)” and greatly affect their survival and health (2). However, the pathogenic mechanism, host-parasite immune interaction, and immune evasion mechanism of *B. schroederi* migration larvae are poorly understood.

The complex mechanism of immune evasion ensures the viability of parasites in the host. Studies have shown that host immune cells are activated mainly through the interaction between Toll-like receptors (TLRs) on their surfaces and excretory secretion (ES) antigens secreted by parasites (4–6). The TLR signaling cascade is responsible for activating and regulating various innate and adaptive immune responses, such as cytokine production, inflammasome activation and assembly, phagocytosis, antigen presentation, and the lymphocyte response (7–9). Therefore, in order to successfully parasitize the host and achieve long-term parasitization, parasites have developed various strategies to appropriately regulate TLR signaling pathways and thus to achieve the optimal immune response (10, 11). Cysteine protease inhibitors (CPIs) are cathepsin modulators that are widely distributed in organisms and participate in various physiological and cellular processes, including immune responses (12, 13). The first CPI found in *Trypanosoma cruzi* was confirmed to play a crucial role in the processes of parasite differentiation and invasion of host cells (14). Subsequently, CPI was identified in several protozoan parasites, including *Trypanosoma brucei*, *Leishmania mexicana*, *Plasmodium falciparum*, and *Toxoplasma gondii* (15–17). Although the biological functions of these proteins are not completely clear, the proteins have been proven to effectively inhibit endogenous cysteine protease, mammalian cathepsin B (CatB), and cathepsin L (CatL), suggesting that they may play a key role in host-parasite interaction. We previously found some CPIs from *B. schroederi* secreting proteins (18). However, it is unclear whether their specific mechanism is similar to other nematodes.

In this study, we cloned and expressed rBsCPI-1, which is highly expressed in *B. schroederi* migratory larvae (ML). We further investigated the signaling pathways involved in rBsCPI-1-mediated regulation of polarization, physiological activities, and antigen presentation response of peripheral blood monocyte-derived macrophages (MDMs), the proliferation and differentiation of CD4+ T cells were also analyzed. The results obtained will help clarify the immune regulation and immune evasion mechanisms of *B. schroederi* and other ascarids and provide a basis for developing immunomodulatory strategies against *B. schroederi* infection.

## Materials and methods

### Experimental animals, parasites and cells

Specific pathogen-free (SPF) 6~8-week-old female Balb/c mice were purchased from Chengdu Dashuo Laboratory Animal Co., Ltd. (n=40 in total). The animal study was reviewed and approved by the Animal Care and Use Committee of Sichuan Agricultural University (SYXK 2019–189). All animal procedures were carried out in accordance with Guide for the Care and Use of Laboratory Animals (National Research Council, Bethesda, MD, USA) and the recommendations of the Animal Research: Reporting of In Vivo Experiments (ARRIVE) guidelines (http://www.nc3rs.org.uk/arrive-guidelines). All applicable institutional and/or national guidelines for the care and use of animals were followed.

*B. schroederi* eggs were separated from giant panda feces collected at the Chengdu Research Base of Giant Panda Breeding. The first-stage (L1) larvae and L2 larvae (migratory larvae, ML) were obtained by incubating the eggs at 28°C in 2.5% formalin. L3 larvae were isolated from the livers and lungs of Balb/c mice 40 days after infection with infectious L2 larvae using Bellman’s method (19). L4-5 larvae and adults were isolated from the intestines of wild giant pandas that died naturally at a nature reserve in Sichuan Province.

Blood samples were taken from mice at one time and used as independent samples for subsequent experiments. Peripheral blood mononuclear cells (PBMCs) were separated with a Mouse Peripheral Blood Mononuclear Cell Separation Solution Kit (TBD, Tianjin, China) according to the instructions. The PBMCs (1 × 10^6^/mL) were cultured in RPMI 1640 (HyClone, Logan, UT, USA) complete medium with 2 mM L-glutamine, 50 ng/mL M-CSF (Solarbio, Beijing, China), and 10% fetal bovine serum (FBS; Sigma, St. Louis, MO, USA) at 37°C and 5% CO_2_ for 48 h. The cultures were rinsed 3 times with RPMI 1640 medium (prewarmed to 37°C). Following rinsing, fresh complete medium was added, and the medium was changed every 2 days until monocytes differentiated and proliferated sufficiently. This required about one week to obtain MDMs (20).

An EasySep™ Mouse CD4+ T cell isolation kit (BioLegend, San Diego, California) was used to purify CD4+ T cells from mouse PBMCs according to the instructions. The sorted cells were cultured with RPMI 1640 complement medium [10% FBS, 100 μg/mL streptomycin and 100 U/mL penicillin (Solarbio, Beijing, China)] at 37°C and 5% CO_2_.

### Analysis of the expression of BsCPIs in *B. schroederi* at different stages

The relative expression of 7 BsCPI genes [CPI-2516, CPI-6422, CPI-3428, CPI-3677, CPI-3427, CPI-2957, CPI-5924; which selected from whole genome of the giant panda roundworm (18) in eggs, L1 larvae, L2 larvae, L3 larvae, L4-5 larvae, and adults of *B. schroederi* was evaluated *via* quantitative real-time PCR (qPCR)]. **Table S1** shows the primer sequences for each detected gene. qPCR was performed using a Roche LightCycler 96 system. Amplifications were conducted with a 20 μL reaction volume containing 10 μL of TB Green Premix Ex Taq™ (Tli RNase H Plus) (TaKaRa, Shiga, Japan), 2 μL of cDNA template (50 ng), 0.8 μL of forward and reverse primers (10 μM), and 6.4 μL of ddH_2_O. The PCR amplification procedure was as follows: 95°C for 10 min; 40 cycles of 95°C for 5 s, 60°C for 30 s; and 95°C for 5 s, 60°C for 60 s, 95°C for 1 s. Transcription levels of the target genes were normalized by subtracting the expression level of GAPDH and then calculating the relative expression using the 2^−ΔΔCt^ method.

### Phylogenetic tree construction

The amino acid sequences of the BsCPI-2957 gene (renamed BsCPI-1, accession number: OM780049) were compared with those CPIs from other organisms. MEGA 7.0 was used to construct the phylogenetic tree. The algorithm was generated into ML (Maximum Likelihood) and NJ (Neighbor-Joining), and the Bootstrap value was set to 1000.

### Cloning and expression of rBsCPI-1

BsCPI-1-specific primers containing the *BamH*I and *EcoR*I restriction sites (bold) were designed according to the coding sequence of BsCPI-1. The primer sequences were 5’-CGC**GGATC**CATGCGCGCGGCAATGC-3 and 5’-CCG**GAATTC**TTAAGAGGTCTCCTTGATTTCTTTTATGGT-3. cDNA extracted from migratory larvae was used as a template to amplify the BsCPI-1 gene. The recombinant expression plasmid pET-32a(+)-BsCPI-1 was constructed and introduced into *Escherichia coli* BL21 (Novagen, USA). rBsCPI-1 was induced with 1 mM isopropyl β-D-1-thiogalactopyranoside (IPTG) at 37°C for 6 h and purified with a nickel (Ni) column. The protein concentration was determined with a BCA Protein Assay Kit (Takara Bio, Dalian, China). The contained endotoxin was removed with a ToxOut™ High Capacity Endotoxin Removal Kit (Smart-Lifesciences, Changzhou, China). At the same time, the endotoxin contented in rBsCPI-1detected by the ToxinSensor™ Chromogenic LAL Endotoxin Assay Kit (GenScript, Nanjing, China) was less than 0.1 EU/mL, it can be considered successfully removed. At the same time, the pET-32a plasmid was directly introduced into *Escherichia coli* BL21 to express the pET-32a protein. And the subsequent procedure was consistent with the expression of rBsCPI-1 recombinant protein.

### Production of antibodies

To generate polyclonal antibodies against rBsCPI-1, approximately 50 μg of rBsCPI-1 protein was intraperitoneally injected into Balb/c mice three times at 7-day intervals, and then mice were anesthetized to collect blood containing specific anti-rBsCPI-1 antibodies in serum. Serum samples collected before protein injection were used as negative sera. And we also collected serum samples from mice 7 days after infection with infectious L2 larvae.

### Immunoblot analysis

Migratory larvae were collected and cultured in RPMI-1640 medium supplemented with 10% FBS, 100 μg/mL streptomycin and 100 U/mL penicillin at 37°C and 5% CO_2_ for 48 h. Collected the culture supernatant containing ES antigens, and PEG2000 was used for concentration before filtration through a 0.22-micron syringe filter (Merck, Darmstadt, Germany). After separation by 12% SDS-PAGE, the ES antigens were transferred to nitrocellulose filter membranes (Biosharp, Guangzhou, China) for Western blot analysis. After blocking nonspecific binding with 5% skim milk, the membranes were incubated with primary antibodies (anti-rBsCPI-1 sera, negative sera) for 1 h at 37°C (1:100 dilution in 5% skim milk). The membranes were then washed three times and incubated with HRP-conjugated rabbit anti-mouse IgG (ABclonal, Wuhan, China) for 1 h at 37°C (diluted 1:5000). Finally, the bound antibodies were detected using ultrasensitive ECL chemiluminescence reagent (Meilunbio, Dalian, China) according to the manufacturer’s instructions.

rBsCPI-1 separated by SDS-PAGE, then transferred to nitrocellulose filter membranes. After blocking nonspecific binding, the membranes were incubated with infective sera. Washed thrice, then incubated with HRP-conjugated rabbit anti-mouse IgG. Finally, the bound antibodies were detected.

MDMs (1 × 10^6^/mL) were treated with 10 µg/mL rBsCPI-1 and equal volume of PBS or pET-32a for 12 h. The cell culture supernatant was discarded and washed with PBS for 3 times. 1 mL RIPA lysis buffer and 1% PMSF (Solarbio) were added to the PBS, pET-32a and rBsCPI-1 group. After lysis was complete, the samples were centrifuged at 12 000×*g* for 5 min to obtain the supernatant. The protein concentration was examined using a BCA assay kit. A total of 50 µg of MDM proteins was separated using SDS-PAGE. The proteins were blotted onto nitrocellulose filter membranes. The membranes were blocked for 2 h at room temperature. Subsequently, the membranes were incubated with anti-NF-κB (1:2000), anti-p38 MAPK (1:2000), anti-ERK1/2 (1:2000), anti-JNK1/2 (1:1000), anti-p-NF-κB (1:1000), anti-p-p38 MAPK (1:2000), anti-p-ERK1/2 (1:2000), and anti-p-JNK1/2 (1:2000) (diluted in blocking buffer, ABclone) antibodies at 4°C overnight. After washing three times, the membranes were incubated with HRP-conjugated secondary antibody for 2 h at room temperature. After washing, ultrasensitive ECL chemiluminescence reagent was dropped to the membranes. The bands were quantified using densitometry and analyzed with ImageJ.

### Analysis of the inhibitory activity of rBsCPI-1 against cysteine proteases

The inhibitory activity of rBsCPI-1 against papain, CatL, and CatB (Sigma) was investigated. rBsCPI-1 and E64 (a positive control for cystatin, purchased from Sigma) were preincubated in assay buffer (100 mM sodium acetate, pH 5.5, 100 mM NaCl, 1 mM DTT) at 37°C for 30 min before addition of CatB, CatL, or papain (0.5 μM). Fluorogenic substrates (Z-Phe-Arg-AMC for papain and CatL and Z-Arg-Arg-AMC for CatB) were added to each reaction mixture, and the mixtures were incubated at 37°C for 1 h. The optical density (OD) was monitored at 405 nm.

### Cell proliferation assay

Approximately 100 µL of MDM suspension (1 × 10^6^/mL) were treated with Concanavalin A (ConA: 10 µg/mL) alone or in the presence of PBS, pET-32a, and different concentrate of rBsCPI-1 (0, 1, 5, 10, 15, 25, and 50 µg/mL) for different times (0, 2, 4, 6, 8, 12, 18, and 24 h). And 10 µL Cell Counting Kit-8 (CCK-8) solution (Meilunbio) was added to each well 4 h before harvesting, the absorbance values were measured at 450 nm.

The sorted CD4+ T cells were resuspended in RPMI 1640 complement medium and labeled with 2 mM CFSE (Sigma-Aldrich) at 37°C in the dark for 10 min. Then, precooled PBS was added to stop the reaction, and the cells were washed three times with PBS. CFSE-labeled CD4+ T cells were resuspended in complete medium at 1×10^5^/mL, and MDMs which treated with 10 µg/mL rBsCPI-1 and equal volume of PBS or pET-32a for 12h were added. The MDMs and CD4+ T cells were co-cultured at 37°C under 5% CO_2_ for 96 h (T cells:MDMs = 1:10). The proliferation of CD4+ T cells was monitored by CFSE dilution using a flow cytometer (FCM) (BD Biosciences, San Jose, CA, USA).

### qPCR analysis

MDMs (1 × 10^6^/mL) were treated with 10 µg/mL rBsCPI-1 and equal volume of PBS or pET-32a for 12 h. Total RNA was extracted from MDMs with a Total RNA Extraction Kit (Solarbio). After synthesizing cDNA from total RNA using a PrimeScript 1st Strand cDNA Synthesis Kit (Takara), the relative expression of TLRs, small Rho GTPases members (Rac1, Cdc42, RhoA), P21-activated kinase 1 (PAK1), proapoptotic (Bax, Fas) and antiapoptotic genes (Bcl-2, Bcl-xL), negative regulators of the TLRs pathway (the suppressor of cytokine signaling (SOCS), toll interacting protein (TOLLIP), single immunoglobulin interleukin-1 receptor related molecules (SIGIRR), interleukin-1 receptor associated kinase-M (IRAK-M), IRAK-2, zinc finger protein A20, and tripartite motif-30α (TRIM-30α)), and MHC-II were analyzed on the same qPCR run as mentioned above.

MDMs (1 × 10^6^/mL) were cultured in the presence or absence of 1 mg/mL TL2.1 (a TLR2-blocking antibody), 7E3 (a TLR4-blocking antibody), Pam3CSK4 (a TLR2-specific activator) (abcam, Shanghai, China), and lipopolysaccharide (LPS, a TLR4-specific activator, Sigma) for 24 h, then treated with 10 µg/mL rBsCPI-1 and equal volume of PBS or pET-32a for 12 h. qPCR was used to analyze the relative expression of M1-subtype macrophage surface markers (IL-12β, IL-6, IL-1β, and iNOS) and M2-subtype macrophage surface markers (CD206, CD163, CD301, Arg1, Ym-1, IL-10) on the same run.

The relative expression of specific markers of Th1 cells (T-bet, IFN-γ), Th2 cells (Gata-3, IL-4), Th17 cells (ROR-γ, IL-17A), Treg cells (Foxp3, TGF-β), and PD-L2, CD80, PD-1, cytotoxic T lymphocyte-associated antigen-4 (CTLA-4) in CD4+ T cells co-cultured with MDMs were analyzed on the same run. **Table S1** shows the primer sequences for each detected gene.

### Cell phagocytosis assay

Briefly, MDMs (1 × 10^6^/mL) were cultured with PBS, pET-32a, and rBsCPI-1 in the presence or absence of TL2.1, 7E3, Pam3CSK4, and LPS. Then, cells were collected and incubated with 1 mg/mL FITC-dextran (Sigma) in RPMI 1640 at 37°C for 1 h. Precooled PBS containing 2% FBS was added to stop the reaction. The cells were washed three times with PBS and resuspended in PBS containing 2% paraformaldehyde to analyze FITC-dextran internalization of MDMs by flow cytometry.

### Cell migration assay

A cell migration assay was performed using a Millicell^®^ insert with 8.0 µm pores (Merck) according to the instructions. MDMs (1 × 10^6^/mL) were cultured with PBS, pET-32a, and rBsCPI-1 in the presence or absence of TL2.1, 7E3, Pam3CSK4, and LPS. And resuspended in RPMI 1640. Then, the cells (200 µL) were seeded into the upper chamber, and the lower chamber was filled with 1200 µL RPMI 1640 complement medium. The cells that migrated through the polycarbonate membrane were determined by crystal violet staining. After repeated washing with PBS, the polycarbonate membrane was decolorized with 33% acetic acid, and the OD value of the eluent was measured at 570 nm.

### Apoptosis assay

Apoptosis was analyzed with an Annexin V-FITC/PI Apoptosis Assay Kit (abcam) according to the manufacturer’s instructions. MDMs (1 × 10^6^/mL) were cultured with PBS, pET-32a, and rBsCPI-1 in the presence or absence of TL2.1, 7E3, Pam3CSK4, and LPS. The cells were resuspended in binding buffer after washing three times with PBS, and added 5 µL Annexin V-FITC with 5 µL PI staining solution. Then incubated in the dark for 10 min. Added 400 μL binding buffer and analyzed immediately by flow cytometry.

### *In Vivo* experiment

A total of 18 SPF 6~8-week-old female Balb/c mice were divided into three groups: the PBS, pET-32a and rBsCPI-1 group. Mice were intraperitoneally injected with 50 μg of rBsCPI-1 and an equal volume of PBS and pET-32a three times at a 7-day interval. Then, the mice were sacrificed 7 days after the last immunization, and PBMCs were collected. The relative expression of M1- and M2-subtype macrophage surface marker molecules; specific markers of Th1, Th2, Th17, and Treg cells; and the activation of the PD-L2/PD-1 and CD80/CTLA-4 pathways were evaluated on the same qPCR run as mentioned above. **Table S1** shows the primer sequences for each detected gene.

### Statistical analysis

All data are expressed as the mean ± SD. Statistical analysis was performed using GraphPad Prism 5.0. ImageJ software was used to quantify the band intensities. The differences between groups were assessed by one-way analysis of variance (ANOVA) in SPSS 26.0 software. *P* ≤ 0.05 was considered to indicate statistical significance.

## Results

### BsCPI-2957 gene was higher expressed in *B. schroederi* migratory larvae

**Figure 1:**
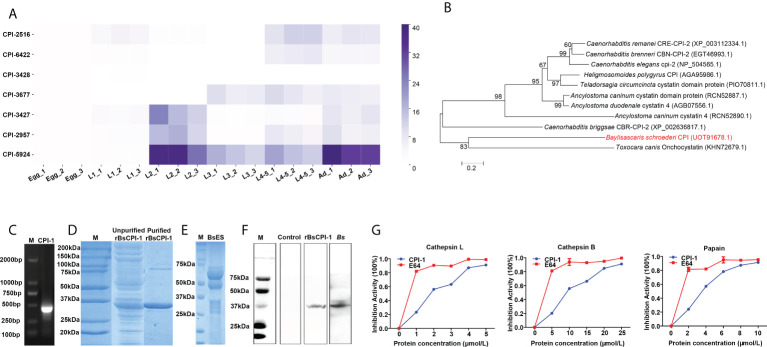
Analysis of the expression and inhibitory activity of rBsCPI-1. **(A)** The relative expression of 7 BsCPIs in *B. schroederi* at different stages was detected by qPCR. **(B)** Phylogenetic tree of CPIs in 11 organisms produced through the ML and NJ method (the red font represents BsCPI-1). **(C)** Amplification of the target band from L2 larval cDNA. M, marker. **(D)** SDS-PAGE gel showing rBsCPI-1. M, protein marker. **(E)** SDS-PAGE gel showing L2 larval ES antigens. M, protein marker; BsES, L2 larval ES proteins. **(F)** Western blot analysis of rBsCPI-1. M, protein marker; rBsCPI-1 was found among L2 larval ES proteins upon probing with anti-rBsCPI-1 serum (rBsCPI-1) but not upon probing with negative (control) serum; and rBsCPI-1 antigen was recognized by mouse serum infected by *B. schroederi* (Bs). **(G)** The inhibitory activity of rBsCPI-1 against CatL, CatB and papain was detected. The activity of the noninhibited enzyme (incubated with 0 μmol/mL rBsCPI-1) was taken as 100% activity (inhibitory activity: 0%). The data are shown as the mean ± SD of 3 repetitions per group.

The results showed that CPI-2516 was higher expressed in L1 larvae, L4-5 larvae and adults; CPI-6422 was higher expressed in L4-5 larvae and adults; CPI-3428 was higher expressed in eggs; CPI-3677 was higher expressed in L3 larvae, L4-5 larvae and adults; CPI-3427 was higher expressed in L2 larvae and adults; CPI-2957 was higher expressed in L2 larvae, L4-5 larvae and adults; and CPI-5924 was higher expressed in L2 larvae, L3 larvae, L4-5 larvae and adults (**Figure 1A**). However, subsequent bioinformatics analysis found that only CPI-2957 had a single conserved sequence of cystatin protein; thus, CPI-2957 was selected for subsequent experiments and renamed CPI-1. And phylogenetic analysis showed that BsCPI-1 was most closely related to *Toxocara canis* onchocystatin (**Figure 1B**).

### Expression and enzyme inhibitory activity analysis of rBsCPI-1

An amplified fragment of the BsCPI-1 gene was successfully obtained from the cDNA of migratory larvae with specific primers, and the correct size was 429 bp (**Figure 1C**). In SDS-PAGE analysis, purified rBsCPI-1 was clearly visible as a single band with an approximate molecular weight of 34.53 kDa, consistent with the predicted size (rBsCPI-1 was 16.53 kDa, and the His-tag protein was 18 kDa) (**Figure 1D**). The Western blot results showed a single band at 34.53 kDa after incubation of ES antigens (**Figure 1E**) with anti-rBsCPI-1 immune serum, but no band was visible after incubation with negative serum. Therefore, the results proved that rBsCPI-1 was a component of ES antigens. Meanwhile, rBsCPI-1 antigen was recognized by serum from mouse which infected with *B. schroederi* (**Figure 1F**). CPIs can inhibit the activity of cathepsin and papain *via* strong binding. E64 is an irreversible, potent, highly selective cysteine protease inhibitor with high purity and stability, and completely inhibited Cat L, Cat B, and papain. In this study, the inhibitory activity of rBsCPI-1 on CatL, CatB, and papain was evaluated. The results showed that rBsCPI-1 effectively inhibited Cat L, Cat B, and papain in a dose-dependent manner. Because the purity and stability of rBsCPI-1 are not as good as E64, the inhibitory effect of rBsCPI-1 on protease is weaker than E64 (**Figure 1G**).

### rBsCPI-1 regulates the TLRs pathway activation

**Figure 2:**
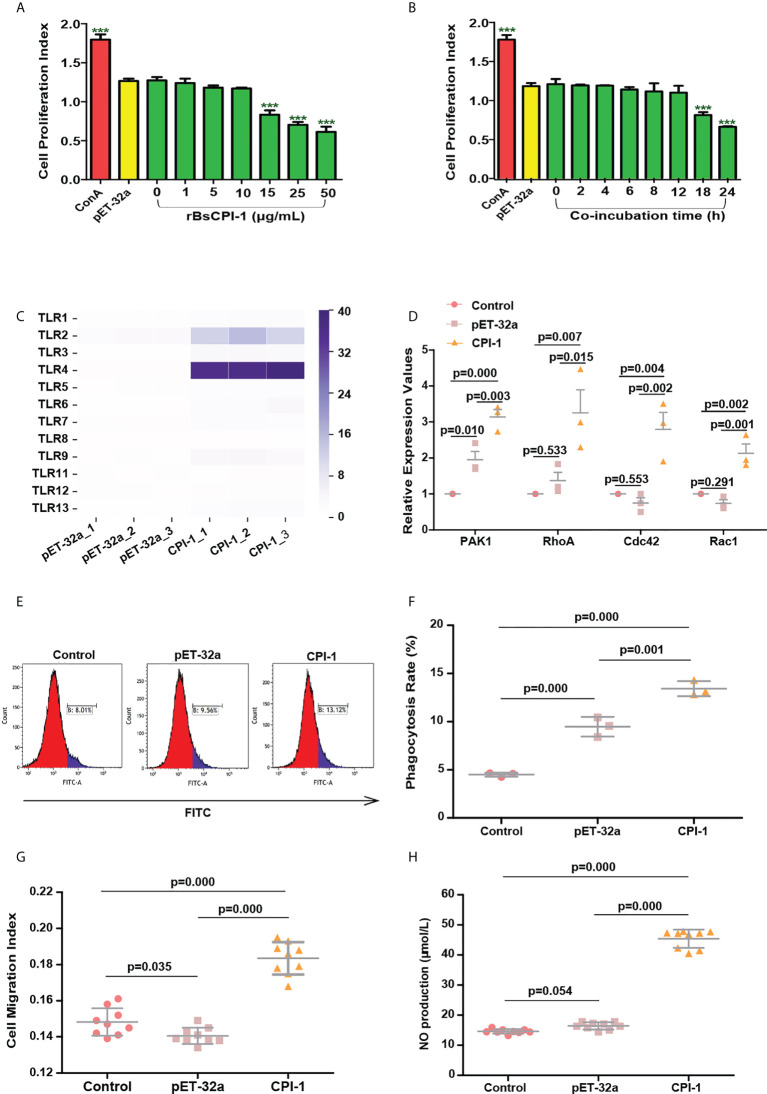
rBsCPI-1 activates the TLR2/4-small Rho GTPases-PAK1 pathway and affects the phagocytosis and migration of MDMs. **(A, B)** rBsCPI-1 effects the proliferation of MDMs. Proliferation was measured by CCK-8 assay after MDMs stimulation with ConA along or combined with PBS, pET-32a and rBsCPI-1. The OD_450_ values were considered the cell proliferation index. ^***^ *P* ≤ 0.001 for other groups vs. the 0 μg/mL group or for other groups vs. the 0 h group. **(C)** rBsCPI-1 increased the relative expression of TLR2 and TLR4 on MDMs. **(D)** rBsCPI-1 affected the relative expression of the RhoA/Rac1/Cdc42-PAK1 pathway in MDMs. **(E)** Phagocytosis of MDMs was determined by analysis of the phagocytosis of FITC-dextran using FCM. **(F)** The percentage of FITC+ cells presented is representative of three independent experiments. **(G)** Random migration was determined by using a Millicell^®^ insert with 8.0 µm pores, and the OD_570_ values were considered the migration index. **(H)** The NO concentration was measured and converted to micromoles per liter (µmol/L) using a standard curve. The data are shown as the mean ± SD of 3 repetitions per group.

As shown in **Figure 2A**, the proliferation of MDMs decreased gradually as the concentration of rBsCPI-1 increased; when the concentration reached 15 μg/mL, proliferation was significantly inhibited (*P* ≤ 0.001). In addition, with increasing co-incubation time, the proliferation of MDMs decreased gradually; when the co-incubation time reached 18 h, proliferation was significantly inhibited (*P* ≤ 0.001) (**Figure 2B**). Therefore, to exclude the influence of cell proliferation on subsequent experimental results, 10 μg/mL and 12 h were selected as the optimal concentration and time for co-incubation of rBsCPI-1 with MDMs.

**Figure 3:**
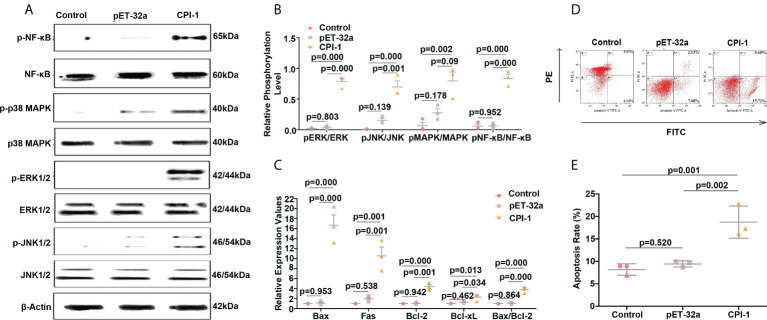
rBsCPI-1 activates the MAPK and NF-κB pathways and affects the apoptosis of MDMs. **(A, B)** Western blot was performed to detect the activation of the MAPK and NF-κB signaling pathways. A representative Western blot is shown in **(A)**, and a graph of the quantified band density is shown in **(B)**. **(C)** qPCR analysis of the transcript levels of apoptosis-related genes. **(D)** Apoptosis of MDMs was determined by staining with annexin V and PI using FCM. **(E)** The total apoptosis rate presented is representative of three independent experiments. The data are shown as the mean ± SD of 3 repetitions per group.

The transcript levels of TLR1, TLR2, TLR3, TLR4, TLR5, TLR6, TLR7, TLR9, TLR12, and TLR13 were significantly higher in the rBsCPI-1 group than in the control group and the pET-32a group, and the differences in TLR2 and TLR4 were the most significant (**Figure 2C**). Therefore, we speculate that rBsCPI-1 can play an immunoregulatory role *via* recognition by TLR2 and/or TLR4 on the surfaces of MDMs. Then, we further analyzed the activation of downstream signaling pathways. The small Rho GTPases (Rac1, Cdc42, RhoA)-PAK1 pathway was identified to play vital roles in cytoskeletal remodeling, cell migration, phagocytosis, and apoptosis (21). The experimental results showed that the transcript levels of Rac1, Cdc42, RhoA, and PAK1 were significantly higher in the rBsCPI-1 group than in the control group and the pET-32a group (**Figure 2D**). NF-κB and MAPK pathway are important transmitters of signals from the cell surface to the nucleus (22). Our results showed that the rBsCPI-1 group exhibited significantly higher phosphorylation levels of NF-κB, p38 MAPK, JNK1/2, and ERK1/2 compared with the control group and the pET-32a group (**Figures 3A, B**).

### rBsCPI-1 regulates the physiological activities of MDMs

As shown in **Figures 2E, F**, phagocytosis of MDMs was significantly greater (*P* ≤ 0.001) in the pET-32a group and the rBsCPI-1 group than in the control group, and rBsCPI-1 induced significantly more phagocytosis of MDMs than pET-32a (*P* ≤ 0.001). The experimental results showed that MDM migration was significantly greater in the rBsCPI-1 group than in the control group and the pET-32a group (*P* ≤ 0.001) (**Figure 2G**). The experimental results showed that rBsCPI-1 induced MDMs to produce high levels of NO, and the concentration in the rBsCPI-1 group was significantly higher than those in the control group and the pET-32a group (*P* ≤ 0.001) (**Figure 2H**). Meanwhile, rBsCPI-1 caused the transcript levels of Bax, Fas, Bcl-2, and Bcl-xL to be significantly higher than those in the control group. In addition, the Bax/Bcl-2 ratio was significantly higher in the rBsCPI-1 group than in the control group (*P* ≤ 0.001) (**Figure 3C**). Therefore, rBsCPI-1 induced MDM apoptosis. The FCM results also showed that the total apoptosis rate (early apoptosis rate + late apoptosis rate) of MDMs was significantly higher in the rBsCPI-1 group than in the control group (*P* ≤ 0.001) and the pET-32a group (*P* ≤ 0.01) (**Figures 3D, E**).

### rBsCPI-1 regulates the phagocytosis, migration and apoptosis of MDMs through TLR2/4

**Figure 4:**
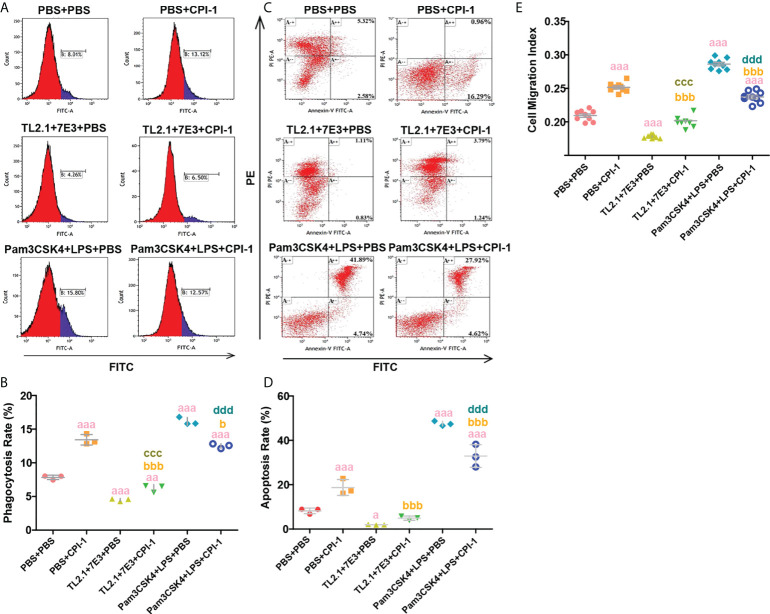
Blocking and activation experiments were performed to analyze TLR2/4 directly affecting phagocytosis, apoptosis and migration. **(A)** Phagocytosis of MDMs was determined by analysis of the phagocytosis of FITC-dextran using FCM. **(B)** The percentage of FITC+ cells presented is representative of three independent experiments. **(C)** Apoptosis of MDMs was determined by staining with annexin V and PI using FCM. **(D)** The total apoptosis rate presented is representative of three independent experiments. **(E)** Random migration was determined by using a Millicell^®^ insert with 8.0 µm pores, and the OD_570_ values were considered the migration index. The data are shown as the mean ± SD of 3 repetitions per group. ^a^ *P* ≤ 0.05, ^aa^ *P* ≤ 0.01, and ^aaa^ *P* ≤ 0.001 vs. the control group (PBS + PBS); ^b^ *P* ≤ 0.05, ^bb^ *P* ≤ 0.01, and ^bbb^ *P* ≤ 0.001 for the TL2.1 + 7E3 + rBsCPI-1 group or Pam3CSK4 + LPS + rBsCPI-1 group vs. the PBS + rBsCPI-1 group; ^c^ *P* ≤ 0.05, ^cc^ *P* ≤ 0.01, and ^ccc^ *P* ≤ 0.001 for the TL2.1 + 7E3 + rBsCPI-1 group vs. the TL2.1 + 7E3 + PBS group; ^d^ *P* ≤ 0.05, ^dd^ *P* ≤ 0.01, and ^ddd^ *P* ≤ 0.001 for the Pam3CSK4 + LPS + rBsCPI-1 group vs. the Pam3CSK4 + LPS + PBS group.

The experimental results showed that pretreatment with TL2.1 + 7E3 significantly decreased the phagocytosis of MDMs (*P* ≤ 0.001); the phagocytosis increased after rBsCPI-1 was added but remained lower than the level in the control group (*P* ≤ 0.01). This suggested that the increased phagocytosis of MDMs induced by rBsCPI-1 required the participation of TLR2 and TLR4. In addition, Pam3CSK4 + LPS pretreatment significantly increased MDM phagocytosis (*P* ≤ 0.001), while the addition of rBsCPI-1 significantly decreased it (*P* ≤ 0.001) (**Figures 4A, B**). Therefore, we speculate that rBsCPI-1 bidirectionally regulates the phagocytosis of MDMs through TLR2 and/or TLR4. We also found that rBsCPI-1 increased the migration of MDMs (*P* ≤ 0.001). When TLR2 and TLR4 were blocked with blocking antibodies, rBsCPI-1 did not significantly promote MDM migration. TLR2 and TLR4 activation significantly increased the migration of MDMs (*P* ≤ 0.001), while the addition of rBsCPI-1 significantly decreased it (*P* ≤ 0.001). Therefore, rBsCPI-1 also plays a significant two-way regulatory role in the migration of MDMs through TLR2 and/or TLR4 (**Figure 4E**). At the same time, TL2.1 + 7E3 pretreatment significantly reduced the apoptosis rate of MDMs (*P* ≤ 0.05); however, after co-incubation with rBsCPI-1, there was no significant difference in the apoptosis rate compared with that of the control group (*P* > 0.05). In addition, pretreatment with Pam3CSK4 + LPS significantly increased the apoptosis rate (*P* ≤ 0.001). After rBsCPI-1 was added, the apoptosis rate decreased significantly (*P* ≤ 0.001) (**Figures 4C, D**). Therefore, we speculate that rBsCPI-1 significantly increases the apoptosis rate of MDMs. When MDM apoptosis is significantly induced, rBsCPI-1 exerts an antiapoptotic effect.

### rBsCPI-1 regulates the polarization of MDMs through TLR2/4

**Figure 5:**
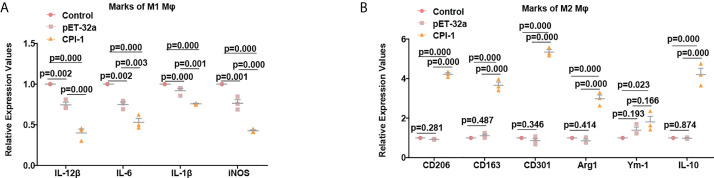
rBsCPI-1 affects the polarization of MDMs. qPCR analysis of the transcript levels of M1 **(A)** and M2 **(B)** macrophage surface marker molecules. The data are shown as the mean ± SD of 3 repetitions per group.

**Figure 6:**
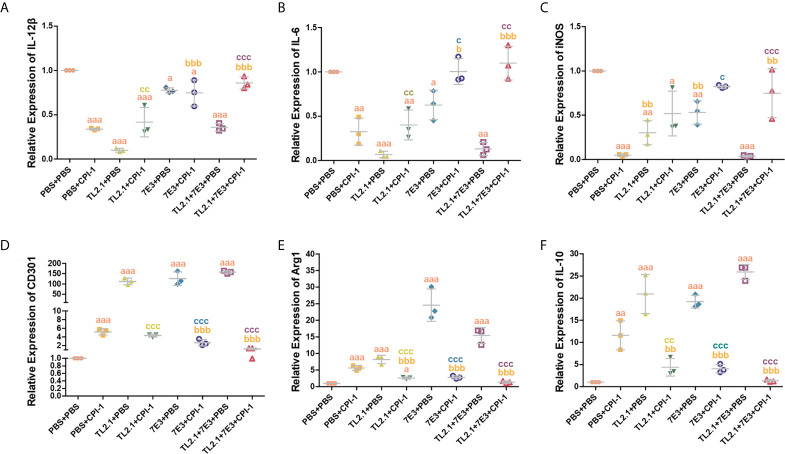
Blocking experiments were performed to analyze TLR2/4 directly affecting the polarization of MDMs. qPCR analysis of the transcript levels of M1 macrophage surface marker molecules **(A–C)** and M2 macrophage surface marker molecules **(D–F)**. The data are shown as the mean ± SD of 3 repetitions per group. ^a^ *P* ≤ 0.05, ^aa^ *P* ≤ 0.01, and ^aaa^ *P* ≤ 0.001 vs. the control group (PBS + PBS); ^b^ *P* ≤ 0.05, ^bb^ *P* ≤ 0.01, and ^bbb^ *P* ≤ 0.001 for the TL2.1+ rBsCPI-1, 7E3 + rBsCPI-1, or TL2.1 + 7E3 + rBsCPI-1 group vs. the PBS + rBsCPI-1 group; ^c^ *P* ≤ 0.05, ^cc^ *P* ≤ 0.01, and ^ccc^ *P* ≤ 0.001 for the TL2.1 + rBsCPI-1 group vs. the TL2.1 + PBS group, the 7E3 + rBsCPI-1 group vs. the 7E3 + PBS group, or the TL2.1 + 7E3 + rBsCPI-1 group vs. the TL2.1 + 7E3 + PBS group.

**Figure 7:**
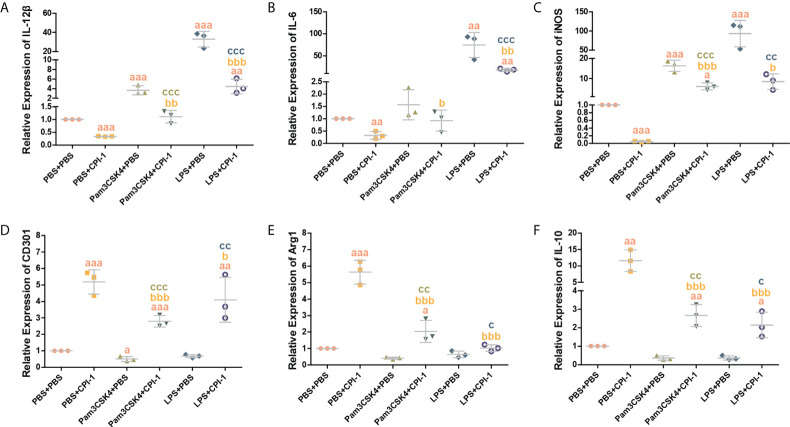
Activation experiments were performed to analyze TLR2/4 directly affecting the polarization of MDMs. qPCR analysis of the transcript levels of M1 macrophage surface marker molecules **(A–C)** and M2 macrophage surface marker molecules **(D–F)**. The data are shown as the mean ± SD of 3 repetitions per group. ^a^ *P* ≤ 0.05, ^aa^ *P* ≤ 0.01, and ^aaa^ *P* ≤ 0.001 vs. the control group (PBS + PBS); ^b^ *P* ≤ 0.05, ^bb^ *P* ≤ 0.01, and ^bbb^ *P* ≤ 0.001 for the Pam3CSK4 + rBsCPI-1, or LPS + rBsCPI-1 group vs. the PBS + rBsCPI-1 group; ^c^ *P* ≤ 0.05, ^cc^ *P* ≤ 0.01, and ^ccc^ *P* ≤ 0.001 for the Pam3CSK4 + rBsCPI-1 group vs. the Pam3CSK4 + PBS group or the LPS + rBsCPI-1 group vs. the LPS + PBS group.

The transcript levels of the M1 macrophage surface markers IL-12β, IL-6, IL-1β, and iNOS were significantly lower in the rBsCPI-1 group than in the control group and the pET-32a group, while those of the M2 macrophage surface markers CD206, CD163, CD301, Arg1, Ym-1, and IL-10 were significantly higher in the rBsCPI-1 group. In summary, rBsCPI-1 induced MDMs to polarize to the M2 subtype, thereby exerting an immunosuppressive effect (**Figure 5**). And the results of TLR2/4 blocking (**Figure 6**) and activation (**Figure 7**) experiments showed that TL2.1 and 7E3 induced polarization of MDMs to the M2 subtype, Pam3CSK4 and LPS induced polarization of MDMs to the M1 subtype, and rBsCPI-1 induced polarization of MDMs to the M2 subtype. When TLR2 was blocked, rBsCPI-1 had no significant regulatory effects on the expression of CD301, and IL-10, while when TLR4 was blocked, rBsCPI-1 had no significant regulatory effects on the expression of IL-6, iNOS, CD301, Arg1, and IL-10. When TLR2 and TLR4 were both blocked, the expression levels of the M1 and M2 macrophage markers did not significantly differ from those in the control group.

**Figure 8:**
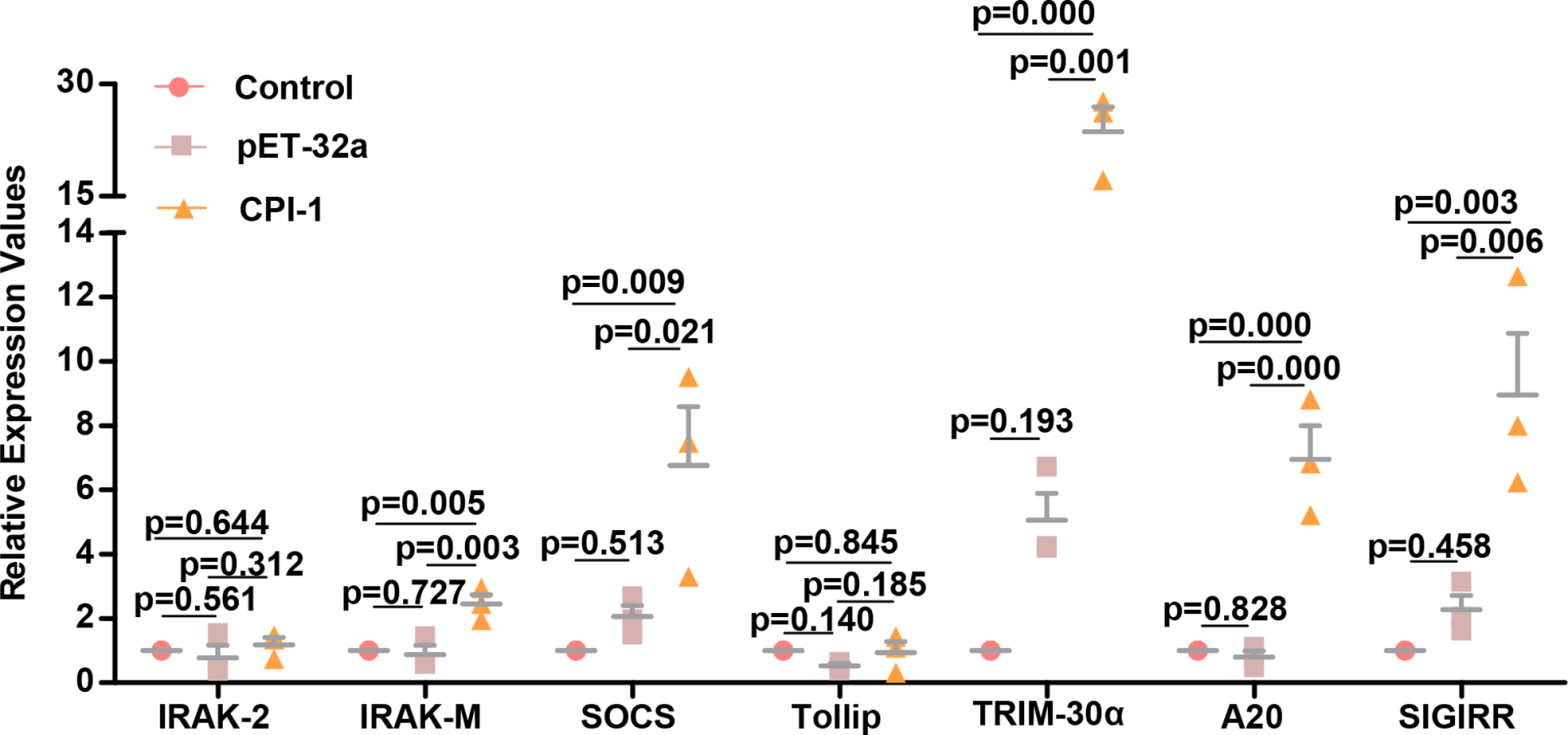
qPCR analysis of the relative expression of negative regulators of the TLR signaling pathway. The data are shown as the mean ± SD of 3 repetitions per group.

Since rBsCPI-1 inhibited the excessive activation of TLRs induced by Pam3CSK4 and LPS, we speculated that negative regulators might also play an essential role in this regulatory process. The qPCR results are shown in **Figure 8**. rBsCPI-1 increased the transcript levels of IRAK-M, SOCS, TRIM-30α, A20, and SIGIRR significantly higher than those in the control group and the pET-32a group. Therefore, rBsCPI-1 can activate the expression of negative regulators and inhibit overexpression of TLR2/TLR4 signal pathway to achieve the optimal immune response.

### rBsCPI-1 regulates T cell proliferation and activation

**Figure 9:**
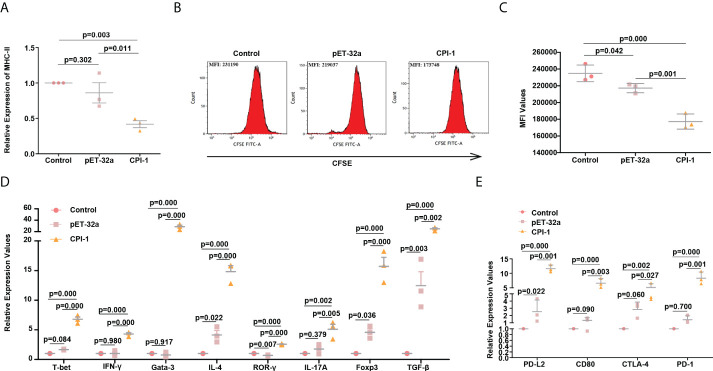
rBsCPI-1 affects the antigen presentation process of MDMs and the proliferation and activation of CD4+ T cells. **(A)** qPCR analysis of the relative expression of MHC-II. **(B)** FCM was performed to detect the mean fluorescence intensity (MFI) of CFSE-labeled CD4+ T cells co-incubated with MDMs which treated with PBS, pET-32a, and rBsCPI-1. **(C)** The FCM data presented are representative of three independent experiments. **(D)** qPCR analysis of the relative expression of surface marker molecules of Th1 cells (T-bet and IFN-γ), Th2 cells (Gata-3 and IL-4), Th17 cells (ROR-γ and IL-17A), and Treg cells (Foxp3 and TGF-β). **(E)** qPCR analysis of the activation of PD-L2/PD-1 and the CTLA-4/CD80 pathway between MDMs and CD4+ T cells.

MHC-II molecules are mainly involved in the presentation of exogenous antigens. These molecules primarily present processed antigen fragments to CD4+ T cells in the initial stage of the immune response (23). Our results showed that the induction of MHC-II molecules was significantly decreased in the rBsCPI-1 group than in the control group and the pET-32a group (*P* ≤ 0.01); therefore, rBsCPI-1 can significantly inhibit the antigen presentation response of MDMs (**Figure 9A**). CD4+ T cells receive the antigens presented by MDMs and subsequently activate the adaptive immune response (23). Therefore, we speculated that rBsCPI-1 might regulate the adaptive immune response by regulating the proliferation and activation of CD4+ T cells. As shown in **Figures 9B, C**, the changes in the mean fluorescence intensity (MFI) indicated that rBsCPI-1 significantly inhibited the proliferation of T cells (*P* ≤ 0.001). CD4+ T cells sensitized by antigens can selectively differentiate into Th1, Th2, Th17, and Treg cells in the local microenvironment (24). Therefore, we used qPCR to detect the expression levels of the Th1 cell surface markers T-bet and IFN-γ, the Th2 cell surface markers Gata-3 and IL-4, the Th17 cell surface markers ROR-γ and IL-17A, and the Treg cell surface markers Foxp3 and TGF-β. The results are shown in **Figure 9D**. rBsCPI-1 induced CD4+ T cells to differentiate into Th1, Th2, Th17, and Treg cells, but the expression levels of Th2 cell surface markers were significantly higher than those of Th1 cell surface markers. In addition, the expression levels of Treg cell surface markers were significantly higher than those of Th17 cell surface markers. Therefore, rBsCPI-1 induces the host to produce a Th1/Th2 mixed immune response dominated by Th2 cells. In addition, although Th17 cells participate in the inflammatory response, Treg cells exert a more significant immunosuppressive effect, thus inhibiting the excessive inflammatory response.

Since rBsCPI-1 can regulate antigen presentation and CD4+ T cell differentiation by regulating the expression of MHC-II molecules on the surface of MDMs, we speculated that the PD-L2/PD-1 and CD80/CTLA-4 pathways between MDMs and CD4+ T cells also played an important role in the immune regulation of rBsCPI-1. The qPCR results showed that the expression levels of PD-L2 and CD80 on the surface of MDMs were significantly higher than those in the control group and the pET-32a group. Similarly, the expression levels of PD-1 and CTLA-4 on the surface of CD4+ T cells were also significantly higher than those in the control group and the pET-32a group (**Figure 9E**).

**Figure 10:**
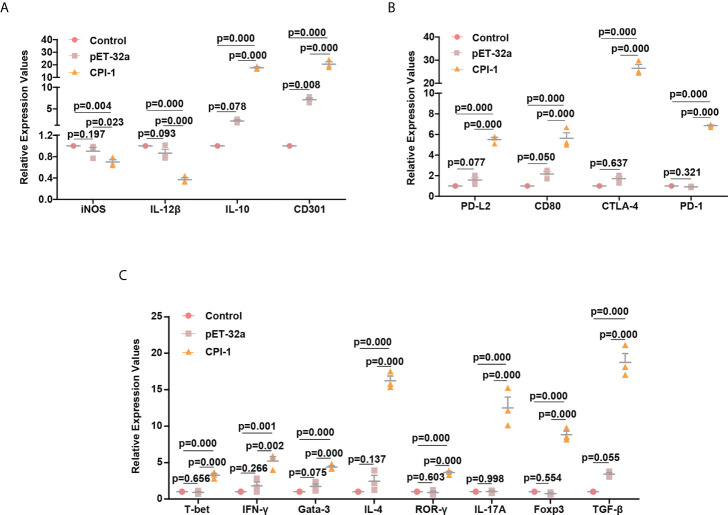
qPCR analysis of the polarization of MDMs and the differentiation of CD4+ T cells *in vitro*. The relative expression of M1 macrophage surface marker molecules (iNOS and IL-12β) and M2 macrophage surface marker molecules (CD301 and IL-10) **(A)**; regulators of the immunoregulatory pathway (PD-L2, PD-1, CD80, and CTLA-4) **(B)**; and cell surface marker molecules for Th1 cells (T-bet and IFN-γ), Th2 cells (Gata-3 and IL-4), Th17 cells (ROR-γ and IL-17A), and Treg cells (Foxp3 and TGF-β) **(C)** were detected by qPCR. The data are shown as the mean ± SD of 3 repetitions per group.

We conducted *in vivo* experiments to verify that rBsCPI-1 regulates the polarization of macrophages and the differentiation of CD4+ T cells. The results are shown in **Figure 10**. Consistent with the results of the cell experiments, rBsCPI-1 induced macrophages to polarize to the M2 subtype and induced a Th1/Th2 mixed immune response dominated by Th2 cells. In addition, the high expression of Treg cells played an immunosuppressive role. Similarly, the PD-L2/PD-1 and CD80/CTLA-4 pathways were also activated, which further inhibited an excessive inflammatory response.

## Discussion

The cathepsins are a class of proteases mainly found in animal cells. In immune cells, cathepsins are involved in antigen degradation and presentation and are pivotal enzymes that initiate the immune response (25). CPIs are natural inhibitors of cathepsins that play essential regulatory roles in many cellular events (26–28). In this study, we cloned, expressed, and identified CPI that is higher expressed in *B. schroederi* migratory larvae based on the whole genome sequence of *B. schroederi* (29). According to sequence similarity and phylogenetic analyses, BsCPI-1 is a member of the cystatin superfamily. Meanwhile, rBsCPI-1 has enzyme inhibitory activity and can significantly inhibit the enzyme activities of papain-like cysteine protease, CatB, and CatL. Western blot further confirmed that rBsCPI-1 is an essential component of ES antigens. Thus, rBsCPI-1 can be secreted by *B. schroederi* and diffuse into the host environment, thereby challenging host immunity by regulating host immune cell functions.

Giant panda species is greatly rare and precious, and previous studies have confirmed that mice infected with *B. schroederi* infectious eggs, migratory larvae penetrate the intestinal wall, then migrate to the other organs for parasitism (30), thus we chose mice as the infective model for the consideration of animal welfare. Macrophages, as multifunctional phagocytes, are rapidly recruited during parasite infection and significantly enhance the host’s innate immune response against the parasites (31). In this study, we found that TLRs, which are critical components of the innate immune system, play a vital role in rBsCPI-1 recognition by MDMs; furthermore, we found that TLR2 and TLR4 have the most significant effects. The members of small Rho GTPase family are located at the central control regulatory site of the pathway, and are influenced by the upstream pathway and then act on the downstream pathway (32). PAK1 can transduce the signals transmitted by the small Rho GTPase family members Cdc42, Rac1, and RhoA to downstream signaling pathways and participate in pleiotropic physiological processes by connecting multiple signaling pathways (20, 21). Therefore, we speculated that rBsCPI-1 may participate in regulating migration, phagocytosis, and other physiological activities related to the cytoskeletal dynamics of MDMs through the Cdc42/Rac1/RhoA-PAK1 signaling pathway. The results were consistent with our expectations: we found that rBsCPI-1 can significantly activate the Cdc42/Rac1/RhoA-PAK1 signaling pathway and induce significant increases in the phagocytosis and migration of MDMs. Although the general view is that TLRs do not act as phagocytic receptors, studies have shown that the TLR signaling pathway can change the phagocytosis of host cells (33, 34). In addition, some studies have confirmed that TLRs can mediate the rearrangement of the macrophage cytoskeleton and promote the diffusion of macrophages to participate in the process of migration (35). Therefore, we speculated that rBsCPI-1 can regulate the phagocytosis and migration of MDMs through the TLR signaling pathway. Blocking and activation experiments showed that rBsCPI-1 cannot significantly regulate the phagocytosis and migration of MDMs when TLR2 and TLR4 are blocked. Our results suggested that rBsCPI-1 regulates the phagocytosis and migration of MDMs through the TLR2/TLR4-Rac1/Cdc42/RhoA-PAK1 signaling pathway.

Notably, many studies have proven that PAK1 mainly affects the MAPK, NF-κB, and other downstream pathways to exert biological effects (22, 36–38). Our Western blot analyses confirmed that rBsCPI-1 induces the phosphorylation of MAPK subfamily members (ERK1/2, JNK1/2, p38 MAPK) and NF-κB, thereby activating the MAPK and NF-κB pathways. The NF-κB and MAPK signal transduction pathways can also regulate apoptosis through various pathways and have different regulatory effects according to the stimulation conditions (39–42). Apoptosis is widely used by parasites as an effective method to weaken the host’s immune response. This study confirmed that rBsCPI-1 induces high expression of antiapoptotic and proapoptotic genes but the proapoptotic response remains dominant. Therefore, rBsCPI-1 induces apoptosis of MDMs. In addition, blocking and activation experiments confirmed that TLR2/4 play a direct role in inducing apoptosis. In short, the existing evidence shows that rBsCPI-1 can mediate apoptosis through the TLR2/4-PAK-1-NF-κB/MAPK signaling pathway, but whether other apoptotic signaling pathways are activated under the influence of rBsCPI-1 remains to be explored.

Macrophages are members of a highly heterogeneous cell population that exhibit different phenotypes and functions in the complex microenvironment (43, 44). In this study, the transcript levels of typical marker molecules on the surfaces of M1 (classically activated) and M2 (alternatively activated) macrophages were detected by qPCR. The results showed that rBsCPI-1 induces macrophages to participate in alternative activation to inhibit the host immune response. The activation of macrophages is induced and regulated by TLRs (45). To verify whether rBsCPI-1 regulates the polarization of MDMs through TLR2 and/or TLR4, we conducted blocking and activation experiments. We found that rBsCPI-1 fails to induce polarization of MDMs when TLR2 and TLR4 are blocked. In addition, TLR2 or TLR4 activation significantly induces MDM polarization to the M1 subtype, and adding rBsCPI-1 induces polarization to the M2 subtype to a certain extent. This result indicated that TLR2 and TLR4 are involved in the polarization of MDMs induced by rBsCPI-1.

rBsCPI-1 does not evade the response generated by TLR recognition; rather, *B. schroederi* has developed a strategy to use TLRs to suppress the immune response in the host. Excessive activation of TLRs can lead to the destruction of immune homeostasis and undesirable side effects. Therefore, the activation of TLR signaling pathways is strictly regulated (46). As shown in our experimental results, rBsCPI-1 can inhibit the abnormal activation of MDMs caused by the excessive activation of TLR2/4. This process also reflects the negative regulation of rBsCPI-1 on the TLR signaling pathway. Therefore, we also detected negative regulators of the TLR signaling pathway. The results were consistent with expectations, indicating that rBsCPI-1 can induce high expression of negative regulators of the TLR pathway and regulate excessive activation to achieve the best activation state.

In the initial stage of the immune response, MDMs can present processed antigen fragments to CD4+ T cells through MHC-II molecules, thereby regulating the proliferation and activation of T cells (23, 24). After co-incubating MDMs stimulated by rBsCPI-1 with CD4+ T cells, we found that the expression of MHC-II molecules was significantly reduced, indicating that rBsCPI-1 can inhibit the antigen presentation response. Furthermore, the proliferation of CD4+ T cells was significantly inhibited by rBsCPI-1, and a Th1/Th2 mixed immune response dominated by Th2 cells was induced. Treg cells that exert immunosuppressive effects were highly differentiated, which was conducive to the formation of an immune tolerance environment. On the one hand, this facilitates the survival of parasites in the host. On the other hand, it also inhibits the occurrence and development of the inflammatory response in the host. Studies have shown that PD-1/PD-L2-mediated cell failure plays an essential role in cancer and occurs in the contexts of chronic infectious diseases caused by protozoan parasites, such as toxoplasmosis and dermal leishmaniasis (47, 48). The CTLA-4/CD80 pathway is another important negative regulatory pathway of the immune response between macrophages and T cells. This study confirmed that rBsCPI-1 can activate the PD-1/PD-L2 and CTLA-4/CD80 pathways. *In vivo* experiments also confirmed that rBsCPI-1 induces macrophages to polarize to the M2 subtype and induces the host to produce a Th1/Th2 mixed immune response dominated by Th2 cells, while Treg cells are highly differentiated. Moreover, members of the PD-L2/PD-1- and CD80/CTLA-4-mediated immunosuppressive pathways are highly expressed, consistent with the results of the *in vitro* experiments.

In conclusion, after being recognized by TLR2 and TLR4, rBsCPI-1 regulates various physiological activities, such as apoptosis, migration, and phagocytosis, of MDMs through a variety of signaling pathways and induces MDM polarization to the M2 subtype to play an immunosuppressive role. rBsCPI-1 can inhibit the expression of MHC-II molecules, thereby inhibiting the antigen presentation of MDMs and regulating the proliferation and activation of CD4+ T cells. The PD-L2/PD-1 and CD80/CTLA-4 pathways between MDMs and CD4+ T cells are significantly activated. In short, rBsCPI-1 can induce the host to produce a mixed immune response dominated by the immunosuppressive response, which is conducive to the immune evasion of *B. schroederi* migratory larvae in the host.

## Data availability statement

The original contributions presented in the study are included in the article/**Supplementary Material**. Further inquiries can be directed to the corresponding author.

## Ethics statement

The animal study was reviewed and approved by Animal Care and Use Committee of Sichuan Agricultural University (SYXK 2019–189).

## Author contributions

JYX participated in the design of the study, feeding experimental animals, the experiments, statistical analysis, and manuscript writing. LX contributed to sample collection and performed the experiments. GYY participated in the design of the study. XBG, YX, RH, JX and XRP helped in study design. All authors have read and approved the final version of the manuscript.

## Funding

This work was supported by a grant from the Research Fund (Project No. CPF2017-24) for the Chengdu Research of Giant Panda Breeding and the Special funding for postdoctoral research projects in Sichuan Province (Project No. 2122999018). The funder had no role in study design, data collection and analysis, preparation or publication of the manuscript.

## Acknowledgments

The authors extremely grateful to teachers and classmates at the Public Laboratory of Sichuan Province in Sichuan Agricultural University for kindly allowing us to conduct the protein purification experiments and use their fluorescence microscope in their laboratories. We would also like to thank the native English-speaking scientists of Elixigen Company (Huntington Beach, California) for editing our manuscript.

## Conflict of interest

The authors declare that the research was conducted in the absence of any commercial or financial relationships that could be construed as a potential conflict of interest.

## Publisher’s note

All claims expressed in this article are solely those of the authors and do not necessarily represent those of their affiliated organizations, or those of the publisher, the editors and the reviewers. Any product that may be evaluated in this article, or claim that may be made by its manufacturer, is not guaranteed or endorsed by the publisher.
