# Supplementary material for: Characterization of a novel cysteine protease inhibitor in Baylisascaris schroederi migratory larvae and its role in regulating mice immune cell response
Source: Front Immunol. 2022 Aug 29;13:894820. doi: 10.3389/fimmu.2022.894820 (PMC9464942; doi:10.3389/fimmu.2022.894820)
Supplement: Supplementary file 1 [file Table_1.docx]

**Table S1 Primers of the detected genes**

|  | Primers | Sequence | Access Number |
| --- | --- | --- | --- |
| CPI-2957 | Forward | 5’-ATGCGCGCGGCAATGC-3’ | UOT91678.1 |
|  | Reverse | 5’-TTAAGAGGTCTCCTTGATTTCTTTTATGGT -3’ |  |
| CPI-2516 | Forward | 5’-ATGCATCCGAGCATAGTGACG-3’ |  |
|  | Reverse | 5’-TTACGCAATATTTCTGCATTCTTTG-3’ |  |
| CPI-3427 | Forward | 5’-ATGGAGGAGGACCCTCCATC -3’ |  |
|  | Reverse | 5’-CTAACCAATTGATATTGGTCCTTTG-3’ |  |
| CPI-3428 | Forward | 5’-ATGCATTCAGGAAGGTGTGGT -3’ |  |
|  | Reverse | 5’-TTATGCTTCCACTGAGAAAGGTG-3’ |  |
| CPI-3677 | Forward | 5’-ATGAACACCAATAGCGGAGAT-3’ |  |
|  | Reverse | 5’-TTAGACGCTGGCTGAGGTAG-3’ |  |
| CPI-2924 | Forward | 5’-ATGGCAAATATCAATGCGCTTAAT -3’ |  |
|  | Reverse | 5’-TTATGCGAGTACACCGACCG-3’ |  |
| CPI-6422 | Forward | 5’-ATGATAATCGGATACGTTATCCTCAT-3’ |  |
|  | Reverse | 5’-TTATTTCTGCTCACATTTTGAGCTC-3’ |  |
| 18s | Forward | 5’-CTCGTTGGCACATCGTAT-3’ |  |
|  | Reverse | 5’-CCTTCTTATCGTCACCTTCT-3’ |  |
| TLR1 | Forward | 5’-ACTCCTTAACTGACCTTCCT-3’ | AY009154.1 |
|  | Reverse | 5’-TCCACCACTTCTCTTGCTA-3’ |  |
| TLR2 | Forward | 5’-CCTTTTATTCTCTGGGCA-3’ | NM_011905.3 |
|  | Reverse | 5’-GTCAGGTGATGGATGTCG-3’ |  |
| TLR3 | Forward | 5’-GGATTGGTGAGTCTGAAGTA -3’ | AF355152.1 |
|  | Reverse | 5’-GGCTATGTTGTTGTTGCTTA-3’ |  |
| TLR4 | Forward | 5’-AACCAGCTGTATTCCCTC-3’ | NM_021297.3 |
|  | Reverse | 5’-TTTCTCACCCAGTCCTCA-3’ |  |
| TLR5 | Forward | 5’-CGAAGACTGCGATGAAGA-3’ | NM_016928.4 |
|  | Reverse | 5’-CTGGATGTTGGAGATATGGT-3’ |  |
| TLR6 | Forward | 5’-GGACTTAGAATACCTGGATGT-3’ | NM_001384171.1 |
|  | Reverse | 5’-GGAACTTGGCAGCACTTA-3’ |  |
| TLR7 | Forward | 5’-TGGACACGGAAGAGACAA-3’ | AY035889.1 |
|  | Reverse | 5’-CAGATGGTTCAGCCTACG-3’ |  |
| TLR8 | Forward | 5’-GAACATCACTCAGGAAGACT-3’ | AY035890.1 |
|  | Reverse | 5’-TCGGAACACATAGCCTCT-3’ |  |
| TLR9 | Forward | 5’-CAGCCACAACATTCTCAAG-3’ | NM_031178.2 |
|  | Reverse | 5’-CTCCAACAGTAAGTCTACGA-3’ |  |
| TLR11 | Forward | 5’-CATCTTCTCAACCAGTCAAC-3’ | NM_205819.3 |
|  | Reverse | 5’-ACCTCCTTCAATAGCATCTC-3’ |  |
| TLR12 | Forward | 5’-CTGTGTCTACTCTGCTTCC-3’ | NM_205823.2 |
|  | Reverse | 5’-TGCCTGCCTTCTTATCCA-3’ |  |
| TLR13 | Forward | 5’-GCCTTCACTCCTCTAATCAA-3’ | NM_205823.2 |
|  | Reverse | 5’-TCACCGTTCCACTCAGAT-3’ |  |
| NOD1 | Forward | 5’-GCTGAGATGCTGAGAGTG-3’ | AY160222.1 |
|  | Reverse | 5’-CTGTTATGGCTGTGTTCTTC-3’ |  |
| NOD2 | Forward | 5’-CCTTCTTGACCATCCTGAC-3’ | NM_145857.2 |
|  | Reverse | 5’-CTCTGTGCGAACGAACTT-3’ |  |
| NLRP1 | Forward | 5’-ACATCCACATACTGCTCAC-3’ | NM_001004142.2 |
|  | Reverse | 5’-ATCTTCACACCACCATCAC-3’ |  |
| NLRP2 | Forward | 5’-TAACCACACGGACACAGT-3’ | NM_177690.3 |
|  | Reverse | 5’-CCAGACAGAGGCAGAATATC-3’ |  |
| NLRP3 | Forward | 5’-AGACCTCCAAGACCACTAC-3’ | BC116175.1 |
|  | Reverse | 5’-ACATAGCAGCGAAGAACTC-3’ |  |
| NLRP4 | Forward | 5’-GCTCAGTCCTCAGAACCT-3’ | NM_001004194.2 |
|  | Reverse | 5’-GCCACCAACTTCATCTCTT-3’ |  |
| NLRP5 | Forward | 5’-CGAGTAGAGAAGTATGTTACCT-3’ | BC053384.1 |
|  | Reverse | 5’-ATGGATGTGTCAAGCAGAG-3’ |  |
| NLRP6 | Forward | 5’-GGTGAAGGAGAGGAATGC-3’ | NM_133946.2 |
|  | Reverse | 5’-GGATGAACAGTAGGCGATT-3’ |  |
| NLRP12 | Forward | 5’-TTACACTCGGCTTCTCCTA-3’ | NM_001033431.1 |
|  | Reverse | 5’-GTTCTTCGTCTGGCTCAA-3’ |  |
| A20 | Forward | 5’-TATGCCACGAATGCTCAG-3’ | U19463.1 |
|  | Reverse | 5’-CAACGCTCACAGAATCCA-3’ |  |
| IRAK-M | Forward | 5’-TGCCAGAAGAATACATCAGA-3’ | AJ440757.2 |
|  | Reverse | 5’-CAAGAACAGTGGAGAAGGA-3’ |  |
| IRAK-2 | Forward | 5’-CCTCCTCTCCACTCTTCA-3’ | BC085324.1 |
|  | Reverse | 5’-AAGTCTCTGTAGCGTTCTG-3’ |  |
| SOCS | Forward | 5’-AGAGGAAGTGACAGAGGAG-3’ | NM_009895.4 |
|  | Reverse | 5’-ACAAGGCTGACCACATCT-3’ |  |
| Tollip | Forward | 5’-GTGGAGGACGAGTGGTAT-3’ | BC062139.1 |
|  | Reverse | 5’-TTCAAGCACAGAACGGATT-3’ |  |
| SIGIRR | Forward | 5’-CAGAGATTGTGTCCAGTGT-3’ | BC094069.1 |
|  | Reverse | 5’-CGTAGGCATCGTATAACTTC-3’ |  |
| TRIM-30α | Forward | 5’-GAAGTAACCTGTCCTATCTGT-3’ | NM_001357467.1 |
|  | Reverse | 5’-TCATCCTGCCATTCATCAC-3’ |  |
| Bax | Forward | 5’-CTGATGGCAACTTCAACTG-3’ | NM_007527.3 |
|  | Reverse | 5’-ATCTTCTTCCAGATGGTGAG-3’ |  |
| Fas | Forward | 5’-AATCGCCTATGGTTGTTGA-3’ | BC061160.1 |
|  | Reverse | 5’-TGTGTCTTGGATGCTGTC-3’ |  |
| Bcl-2 | Forward | 5’-TCGTGACTTCGCAGAGAT-3’ | NM_009741.5 |
|  | Reverse | 5’-CAGAGACAGCCAGGAGAA-3’ |  |
| Bcl-xL | Forward | 5’-GGAGAGCGTTCAGTGATC-3’ | L35049.1 |
|  | Reverse | 5’-AGGTGGTCATTCAGATAGGT-3’ |  |
| GAPDH | Forward | 5’-TCTCCTGCGACTTCAACA-3’ | AY618199.1 |
|  | Reverse | 5’-TGTAGCCGTATTCATTGTCA-3’ |  |
| PAK1 | Forward | 5’-AGATGATGATGACGATGCTA-3’ | NM_011035.2 |
|  | Reverse | 5’-GCGGAGTGGTGTTATTCT-3’ |  |
| RhoA | Forward | 5’-ATGCTTGCTCATAGTCTTCA-3’ | JN971019.1 |
|  | Reverse | 5’-TTGCCATATCTCTGCCTTC-3’ |  |
| Cdc42 | Forward | 5’-ACAGACTACGACCGCTAA-3’ | U37720.1 |
|  | Reverse | 5’-GCACTCCACATACTTGACA-3’ |  |
| Rac1 | Forward | 5’-CAGACAGTTGGAGACACAT-3’ | NM001347530.1 |
|  | Reverse | 5’-ACGAGGATGATAGGAGTATTG-3’ |  |
| IL-12β | Forward | 5’-GAATGGCGTCTCTGTCTG-3’ | NM_001303244.1 |
|  | Reverse | 5’-GCTGGTGCTGTAGTTCTC-3’ |  |
| IL-6 | Forward | 5’-TCCATCCAGTTGCCTTCT-3’ | M20572.1 |
|  | Reverse | 5’-TAAGCCTCCGACTTGTGA-3’ |  |
| IL-1β | Forward | 5’-CTTCAGGCAGGCAGTATC-3’ | NM_008361.4 |
|  | Reverse | 5’-CAGCAGGTTATCATCATCATC-3’ |  |
| iNOS | Forward | 5’-ACAGCCTCAGAGTCCTTC-3’ | BC062378.1 |
|  | Reverse | 5’-TCACCACCAGCAGTAGTT-3’ |  |
| CD206 | Forward | 5’-TGGAAGAAGAAGTAGCCTATC-3’ | NM_008625.2 |
|  | Reverse | 5’-TGGAGTAGTGGTTGGAGAA-3’ |  |
| CD163 | Forward | 5’-CAGACTGGTTGGAGGAGA-3’ | BC144848.1 |
|  | Reverse | 5’-CTATGTATCGTGAGCAGACTA-3’ |  |
| CD301 | Forward | 5’-AGACAACACCACCTCCAA-3’ | NM_001204252.1 |
|  | Reverse | 5’-CACGACTTCTCAGACTCAG-3’ |  |
| Arg-1 | Forward | 5’-AAGGTCTCTACATCACAGAAG-3’ | NM_007482.3 |
|  | Reverse | 5’-CGAAGCAAGCCAAGGTTA-3’ |  |
| Ym-1 | Forward | 5’-CTCCTCAGAACCGTCAGA-3’ | NM_009892.3 |
|  | Reverse | 5’-CTCCAGTGTAGCCATCCT-3’ |  |
| IL-10 | Forward | 5’-ACTGCTAACCGACTCCTT-3’ | NM_010548.2 |
|  | Reverse | 5’-TCCACTGCCTTGCTCTTA-3’ |  |
| IL-4 | Forward | 5’-TCCTGCTCTTCTTTCTCG-3’ | M29854.1 |
|  | Reverse | 5’-TTCTCCTGTGACCTCGTT-3’ |  |
| IFN-γ | Forward | 5’-TCTGAGACAATGAACGCTAC-3’ | NM_008337.4 |
|  | Reverse | 5’-TTCCACATCTATGCCACT-3’ |  |
| IL-17A | Forward | 5’-CCTCTGTGATCTGGGAAGC-3’ | NM_010552.3 |
|  | Reverse | 5’-CACGAAGCAGTTTGGGAC-3’ |  |
| TGF-β | Forward | 5’-GCAACAACGCCATCTATG-3’ | M13177.1 |
|  | Reverse | 5’-CAAGGTAACGCCAGGAAT-3’ |  |
| T-bet | Forward | 5’-GGAGGTGAATGATGGAGAG-3’ | BC137988.1 |
|  | Reverse | 5’-GGATACTGGTTGGATAGAAGA-3’ |  |
| Gata-3 | Forward | 5’-TCCAGTCCTCATCTCTTCA-3’ | X55123.1 |
|  | Reverse | 5’-GGCGGATAGGTGGTAATG-3’ |  |
| ROR-γ | Forward | 5’-ATTCAGTATGTGGTGGAGTT-3’ | AJ132394.1 |
|  | Reverse | 5’-GTGGTTGTTGGCATTGTAG-3’ |  |
| Foxp3 | Forward | 5’-ATGTTCGCCTACTTCAGAA-3’ | NM_001199348.1 |
|  | Reverse | 5’-TCATCTACGGTCCACACT-3’ |  |
| MHC-II | Forward | 5’-CTCCTCCATCCACTGTCT-3’ | NM_010398.3 |
|  | Reverse | 5’-TGTCTCCTCCTCTTCATCA-3’ |  |
| PD-L2 | Forward | 5’-CAACACCAGCCACATCAG-3’ | NM_021396.2 |
|  | Reverse | 5’-TTCCATCCGACTCAGAGG-3’ |  |
| PD-1 | Forward | 5’-AGGACGACACTCTGAAGG-3’ | NM_008798.3 |
|  | Reverse | 5’-GACAATGGTGGCATATTCTG-3’ |  |
| CD80 | Forward | 5’-ATTACCTGGCATCAATACGA-3’ | BC145843.1 |
|  | Reverse | 5’-GACGACGACTGTTATTACTG-3’ |  |
| CTLA-4 | Forward | 5’-ACGACATTCACAGAGAAGAA-3’ | X05719.1 |
|  | Reverse | 5’-AACAGCAGAGACCAGGAA-3’ |  |
